# Immunomodulatory and Antioxidant Properties of *Ipomoea batatas* Flour and Extracts Obtained by Green Extraction

**DOI:** 10.3390/cimb45090440

**Published:** 2023-08-22

**Authors:** Imane Boukhers, Sylvie Morel, Joelle Kongolo, Romain Domingo, Adrien Servent, Lea Ollier, Hippolyte Kodja, Thomas Petit, Patrick Poucheret

**Affiliations:** 1 Qualisud, Univ Montpellier, Avignon Université, CIRAD, Institut Agro, IRD, Université de La Réunion, 97400 Montpellier, France; imane.boukhers@gmail.com (I.B.); joelle.kongolo@univ-reunion.fr (J.K.); romain.domingo@cirad.fr (R.D.); adrien.servent@cirad.fr (A.S.); lea.ollier@cirad.fr (L.O.); hippolyte.kodja@univ-reunion.fr (H.K.); 2 Laboratoire de Botanique, Phytochimie et Mycologie, CEFE, CNRS-Université de Montpellier-Université Paul-Valéry Montpellier-EPHE-IRD, 34093 Montpellier, France; sylvie.morel@umontpellier.fr; 3 Laboratoire de Chimie des Substances Naturelles et des Sciences des Aliments (LCSNSA), Université de La Réunion, 34093 Sainte-Clotilde, France; thomas.petit@univ-reunion.fr

**Keywords:** orange flesh sweet potato flour, phenolic compounds, beta-carotene, antioxidant activity, anti-inflammatory activity, chronic metabolic diseases

## Abstract

Sweet potato (SP), *Ipomoea batatas* Lam, belongs to the Convolvulaceae family. It produces edible storage roots. Currently, orange varieties contribute to improving food systems and managing vitamin A deficiency. Processing of this food crop into flour allows better conservation. However, nutrition health data regarding SP flour obtained by green extraction remains scarce. In this study, we therefore explored its phytochemistry and its associated bioactivity potential for human health. We analyzed the nutritional composition of orange flesh sweet potato (OFSP) flour and assessed the antioxidant (free radical scavenging) and immunomodulatory (on inflammatory murine macrophages) properties of the extract. More specifically, we measured the impact of OFSP flour extract on mediators such as Nitric Oxide (NO) and the production of pro-inflammatory cytokines such as Interleukin-6 (IL-6), Tumor Necrosis Factor alpha (TNF-alpha), Monocyte Chemoattractant Protein-1 (MCP-1), and Prostaglandin-E2 (PGE-2). Our results indicated significant fiber, mineral, beta-carotene, and polyphenols content in the extracts, and antioxidant and immunomodulatory bioactivities were also demonstrated with a concentration-dependent inhibition of cytokine production. Taken together, our results suggest that *Ipomoea batatas* flour could, in addition to being a good source of energy and beta-carotene provitamin A, constitute a food of interest for the prophylaxis of metabolic diseases associated with an underlying low-grade inflammatory state.

## 1. Introduction

Carbohydrates and more particularly starch represent the main source of energy for a healthy and balanced diet. This polysaccharide is used by vegetals as carbohydrate storage at different levels of the plant. It accounts for nearly 85% of the dry weight of seeds in cereals that contribute to the annual production of starch and that are the main source of food for humans and livestock worldwide [1].

In addition to cereals, starchy roots and tubers are sources of starch. They provide a substantial part of the world’s food supply, and they play a central role in human nutrition, particularly in countries with the lowest rate of cereal self-sufficiency [2].

Asia and Africa are the main producers, with 43% and 33% of the total world production, respectively [3]. Various species and varieties are consumed, but cassava, potatoes, and sweet potatoes account for 90% of the total global yield [3,4].

Sweet potato is native to tropical American regions. It belongs to the genus ipomoea, which belongs to the Convolvulaceae family. It is currently cultivated on all continents, and, as is the case with many tubers and roots, its cultivation is very undemanding [5]. Among the sweet potato varieties, those with orange flesh have been considered the most successful example of staple crop biofortification to remedy vitamin A deficiency. In 2016, a group of scientists received the 30th World Food Prize for the development and promotion of a biofortified orange-fleshed sweet potato [6].

Growing this root vegetable in countries where chronic malnutrition is prevalent has become a strategy to improve nutrition due to its beta-carotene content. Its cultivation is promoted and strategies have been implemented in both Africa and Asia for its integration into the main food systems in order to reduce the prevalence of vitamin A deficiency [7,8,9,10,11]. Indeed, vitamin A deficiency is one of the most important nutritional deficiencies in the world [12]. It can lead to the growth and development of deficits in children, impaired vision, and increased risk of infection [13,14], and it is also responsible for certain types of diseases, such as cognitive disorders [15]. The World Health Organization (WHO) estimates that more than 200 million women and children worldwide are affected [16]. In Mozambique, it is estimated that 34.8% of childhood deaths under the age of 5 are caused by vitamin A deficiency [17]. In addition to the impact on avitaminosis, beta-carotene has other beneficial properties on human health. Studies have established an opposite association between its serum levels and markers of systemic inflammation, insulin resistance, and the dysfunction of beta cells. In addition, a diet rich in beta-carotene has been shown to prevent cardiovascular disease, stroke, and certain types of cancers, and these are just some of the positive health effects [18,19,20,21].

Furthermore, beta-carotene, *Ipomoea batatas* Lam, contains other secondary metabolites and, more specifically, polyphenolic compounds. This phytochemical family of organic molecules is widely present in the plant kingdom. It has been extensively studied and classified into different categories associated with various properties of interest for human health [22]. Research on the bioactive compounds in orange-fleshed sweet potato (OFSP) indicate the presence of phenolic acids and flavonoids. Sweet potato phenolic acids are mainly composed of a mixture of caffeic and caffeoylquinic acid derivatives––namely, caffeic acid, chlorogenic acid, 3,4-di-O-caffeoylquinic acid, 3,5-di-O-caffeoylquinic acid, and 4,5-di-O-caffeoylquinic acid [23,24,25]. Other phenolic acids were also detected, including coumaric acid, salicylic acid, and protocatechuic acid [26]. As for flavonoids, studies indicate the presence of rutin, apigenin, myricetin, quercetin, and kaempferol [27]. These phytochemicals were demonstrated to have anticancer properties as well as positive impacts on neurodegenerative diseases, obesity, type 2 diabetes, and cardiovascular and metabolic diseases, which currently represent the most important causes of death in the world [22,28]. Their health effects were largely attributed to their antioxidant and anti-inflammatory properties [29,30] expressed through a variety of mechanisms, including scavenging free radicals and reducing their production at the mitochondrial level [31]; modulating cell signaling and enzymatic activities [32,33]; reducing inflammatory responses via inflammatory signaling pathways and cytokines modulation [28,34]; insulin resistance prophylaxis; and dyslipidemia and hypertension improvement [35,36], with a direct impact on cardiovascular disease, glycemic control, and metabolic profile [37,38,39].

Therefore, the aim of the present study was to assess the protective potential of a variety of OFSP flours cultivated in France against chronic metabolic diseases. For this purpose, we first evaluated OFSP phytochemistry and physicochemical properties, focusing on resistant starch, mineral matter, fibers, and beta-carotene. Second, we measured the associated antioxidant and immunomodulatory activities through the scavenging of free radicals and the inhibition of the production of inflammation mediators by stimulated inflammatory macrophages. Our objective was to provide scientific data on OFSP flour obtained by the green extraction process in order to support the development of new sustainable food systems centered on energy source plant resources that are associated with health-promoting properties. This is part of a general approach to develop functional foods that provide, beyond basic nutritional needs, physiological benefits that contribute to reducing the risk of chronic metabolic diseases, such as metabolic syndrome and its co-morbidities.

## 2. Materials and Methods

### 2.1. Plant Material

*Ipomoea batatas* tuber samples were collected from a 6-month-old plantation in a region of Southern France (Occitania). Tubers were collected from 27 individuals of 3 different plots on the same plantation (same variety, same pedoclimatic conditions). Identification was performed by a botanist, and a sample was kept in the Department of Pharmacology and Physiopathology of the University of Montpellier under the code IB.OFSP.032.

After collection, tubers were carefully cleaned with distilled water, cut into pieces without being peeled, and directly dried in a hot air oven at 45 °C for 48 h before being grinded. The resulting flour was sieved, packed in opaque bags, and protected from moisture.

### 2.2. Phytochemichal Analysis and Glycemic Index

#### 2.2.1. Determination of Carbohydrates

##### Total Starch

The total starch content was determined by measuring D-glucose after its enzymatic degradation. For this purpose, 500 mg of flour samples were suspended in 30 mL of water. Once solubilized, starch was then hydrolyzed by adding pancreatic alpha-amylase (PAA, which hydrolyzes complex carbohydrates into maltose and small-sized polysaccharides, such as dextrin) and incubated in a water bath at 98 °C for 25 min. A second hydrolysis was performed with amyloglucosidase (AMG, which hydrolyzes starch and small-sized polysaccharides, such as dextrin, into glucose) for 30 min at 60 °C. The colorimetric reaction was performed with glucose oxidase/peroxidase reagent (GOPOD), and the D-glucose (Sigma-Aldricht, Illkirch, France) content was measured with a spectrophotometer (Shimadzu UV 2450 spectrophotometer, Shimaszu, Paris, France) at 510 nm. Total starch content was determined after measurement and subtraction of the amount of simple sugars contained in the sample.

##### Free Sugars

The content of simple sugars was determined by the enzymatic method. Briefly, 50 mg of flour was weighed before the addition of 1 mL of a 5 mN sulfuric acid solution (Sigma-Aldricht, Illkirch, France). Tubes were then submitted to orbital shaking for 1 h at 20 rpm. Samples were then centrifugated at 10,000× *g*. A total of 100 μL of this solution was removed and mixed with 200 μL of amylo-glucosidase, followed by an incubation of 30 min at room temperature. Subsequently, 2.5 mL of GOPOD reagent was added. After 20 min of incubation, the amount of free sugars was determined using a glucose calibration curve. The measurement was carried out at 510 nm.

##### Resistant Starch

The determination of resistant starch (RS) was performed with the K-RAPRS 11/19 Megazyme RS KIT (Sigma-Aldricht, Illkirch, France) following the AOAC 2017.1618 method. Briefly, 100 mg of flour samples were incubated in a linear motion stirred water bath with saturating levels of purified PAA and AMG (Sigma-Aldricht, Illkirch, France) for 4 h at 37 °C. During this time, non-resistant starch was solubilized and hydrolyzed to D-glucose by the combined action of the two enzymes. The reaction was stopped by adding an equal volume of ethanol. RS was recovered as a pellet after centrifugation. The pellet was then dissolved in 1.7 M NaOH, and the starch was hydrolyzed to D-glucose with AMG solution (Sigma-Aldricht, Illkirch, France). The colorimetric reaction was performed with GOPOD reagent, and the measurement of the D-glucose content was performed at 510 nm.

##### Determination of Amylose

The amylose content was determined according to the protocol following the ISO 2020-6647 Standard [40]. Briefly, 100 ± 0.5 mg of dry flour were mixed with 1 mL of ethanol (95% *v*/*v*), and then submitted to an extraction with 9 mL of 2 M NaOH before centrifugation for 10 min at 900 rpm. The sample was then transferred into a 100 mL volumetric flask. Using a pipette, a 5 mL sample aliquot was mixed with 50 mL of water. After the addition of 2 mL of 1 M acetic acid and 2 mL of iodine solution (Sigma-Aldricht, Illkirch, France), the volume was completed up to 100 mL with water. After 10 min of incubation at room temperature, the absorbance was measured at 720 nm to determine the amylose content.

##### Quantification of Insoluble Fiber

Determination was performed according to the Van Soest method [41] on 1 g ± 5 mg of the flour sample. Fiber fractionation of lignocellulosic biomass was performed by successive chemical extractions with neutral and then acidic detergents. For NDFs*, a neutral detergent solution (sodium lauryl sulfate, EDTA; pH 7) (Sigma-Aldricht, Illkirch, France) at boiling temperature with a thermostable α-amylase Termamyl^®^ was used to dissolve easily digested pectins and cell contents, leaving an NDF fiber residue. For ADFs**, an acidic detergent solution (cetyl-trimethylammonium-bromide and H_2_SO_4_) (Sigma-Aldricht, Illkirch, France) was used to dissolve hemicellulose. Finally, for ADL, a 3-h digestion was performed with 72% H_2_SO_4_. Dry insoluble fibers recovered by filtration were quantified gravimetrically.

(*NDF represent Hemicellulose + Cellulose + Lignin; ADF corresponds to cellulose + lignin. **ADL corresponds to lignin, and the results are expressed in percentage of dry matter of flour.)

##### Measurement of the Glycemic Index (GI)

The animals (Wistar rats) were housed in a temperature-controlled room (20–22 °C) with a 12-h dark–light cycle. They were accommodated in three rats per cage, where they were fed with a standard A04 SAFE-Augy-France diet and water ad libitum. The experiments were carried out in accordance with the rules of ethics and animal welfare. The experimental protocol was approved by the Languedoc-Roussillon Ethics Committee (Authorization N-APAFIS#2386-20200101409859 v3).

The glycemic index of OFSP flour was measured in animal experiments on groups of 8 male Wistar rats with an average weight of 318.3 ± 11.6 g. The tests were performed after a 8-h fasting period with solubilized flour. The dose administered corresponded to an equivalent of 2 g of carbohydrate in 2 mL/kg of rat weight. In parallel, another group of 8 rats with an average weight of 320.5 ± 9.7 received 2 g of glucose/kg solubilized in water.

One drop of tail blood sample was taken at 15 min intervals over a period of 3 h to determine blood glucose level with a Contour Bayer Glucometer (Bayer Pharmaceuticals, France). From these data, blood glucose curves were plotted. The GI was calculated by dividing the incremental area under the curve of the test food by the incremental area under the curve of the reference food and then multiplying the result by 100.

### 2.3. Identification and Quantification of Bioactive Phytochemicals

#### 2.3.1. Total Phenolic Content Test

The TPC method was performed on a dry extract of OFSP flour, for which an extraction of potentially bioactive compounds was performed as follows. Briefly, 50 g of flour sample was dispersed in 200 mL of ethanol/water mix (80:20, *v*/*v*) (Sigma-Aldricht, Illkirch, France). The solution was sonicated with an ultrasonic bath Type VWR USC 300 TH for 30 min at 35 °C and then filtered (Extract 1). The retentate was then subjected to a second extraction, which was identical to the first (Extract 2), and then a third extraction with methanol/water (80:20, *v*/*v*) (Extract 3). The supernatants of the three extractions were combined and evaporated under a fume hood until they were dried. This extract was also used for the potential evaluation of antioxidants and anti-inflammatory potential.

Total polyphenols assay was performed with Folin-Ciocalteu reagent according to the method of Morel et al. [42]. The dry extract of OFSP flour was prepared in DMSO (Sigma-Aldricht, Illkirch, France) at 4 mg/mL, and was then diluted in water to be tested at a concentration of 1 mg/mL. A calibration curve generated concentrations ranging from 1.56 to 75 μg/mL of gallic acid. In a 96-well plate, 50 μL of extract, or 50 μL of gallic acid, and 50 μL of distilled water were distributed in triplicate. Then, 50 μL of 10% Folin Ciocalteu reagent and 50 μL of sodium carbonate solution (1 M) were added. After 60 min in the dark, the absorbance was measured on a microplate reader (Thermofisher, Paris, France) at a wavelength of 650 nm. Results were expressed as milligrams of gallic acid equivalents (GAE) per gram of OFSP flour extract.

#### 2.3.2. Identification and Quantification of Carotenoids

Extraction of carotenoids from OFSP flour was performed three times on an ethanol/hexane mix (4:3, *v*/*v*) sample. The residue was separated from the liquid phase by filtration with a filter funnel (pore size #2). The organic phases of the three extractions were transferred to a separating funnel. A total of 10 mL of NaCl was then added to saturate the aqueous phase. This water phase was then discarded. The hexane phase was dried using anhydrous sodium sulfate and filtered before evaporation under vacuum. The carotenoid extracts were dissolved in 1 mL of 80:20 (*v*/*v*) mixture of methyl tert-butyl ether (MTBE) and methanol (Sigma-Aldricht, Illkirch, France) before being analyzed by HPLC.

Carotenoids were analyzed by HPLC using the Agilent 1100 system (Agilent, Massy, France) with a diode array detector. Carotenoids were separated along a C30 column 250 × 4.6 mm i.d., 5 µm (YMC, Tokyo, Japan); the mobile phases were H_2_O as eluent A, methanol as eluent B, and MTBE (methyl-tert-butyl-ether) as eluent C with a gradient. The flow rate was fixed at 1 mL/min, the column temperature was set at 25 °C, and the injection volume was 20 µL. The absorbance was measured at 450 nm. Chromatographic data and UV-Visible spectra were treated using the Agilent Chemstation plus software (ChemStation B.04.0x, Agilent, Paris, France).

#### 2.3.3. Determination of Mineral Materials

Determination of the total amount of mineral matter was performed after fractionation and quantification of the fibers. Briefly, mineralization of the remaining sample was performed at 500 °C in an ash furnace (Thermo Scientific™ Thermolyne™ 6000 series 408, Waltham, MA, USA), and the material obtained was quantified gravimetrically.

### 2.4. Evaluation of the Bioactive Potential

#### 2.4.1. Antioxidant Activity

The antioxidant activity was evaluated in vitro by measuring the NO (Nitric Oxide) scavenging potential, the free radical scavenging capacity with the DPPH (2,2-diphenyl 1-picrylhydrazyl) test, and the antioxidant capacity with the ORAC test.

##### Determination of NO (Nitric Oxide) Scavenging

The flour samples were tested at different concentrations (100, 75, and 50 µg/mL) in order to determine their nitric oxide scavenging capacity. For this purpose, sample solutions were incubated with a volume ratio of 1:1 in 5 mM of sodium nitroprusside solution diluted in HBSS for 2 h under a light source at room temperature. NO was then determined as nitrite using the Griess method and a standard curve of NaNO_2_ (1.56 to 100 µM). The reading was performed at 550 nm, and the results were expressed as a percentage of NO scavenging.

##### DPPH (2,2-Diphenyl-1-picrylhydrazyl) Assay

Antioxidant activity was evaluated using the DPPH assay according to the method of Morel et al. [42]. Extracts were solubilized in DMSO (4 mg/mL) before being diluted in absolute ethanol to reach a concentration of 1 mg/mL. A standard curve of Trolox was realized and plotted (75, 50, 25, 12.5 µM). Ethanol was used as the blank and chlorogenic acid (0.01 mg/mL) was used as the positive control.

In a 96-well plate, 100 µL of the positive control or the extract was added into each well. The test was performed in triplicate for each extract. A total of 75 µL of absolute ethanol and 25 µL of extemporaneously prepared DPPH solution (0.4 mg/mL) were introduced into each well. The plate was incubated for 30 min at room temperature and protected from light. The absorbance was read at 550 nm with a microplate reader (MDS Inc., Toronto, ON, Canada). Results were expressed as Trolox equivalents (TE µmoles per gram of dry extract) and as a percentage of inhibition (% inhibition).

##### ORAC (Oxygen Radical Absorbance Capacity) Assay

The ORAC (Oxygen Radical Absorbance Capacity) assays were performed in 96-well opaque polypropylene plates, as previously described [42].

Samples were solubilized in DMSO at a concentration of 1 mg/mL before being diluted to 25 µg/mL using phosphate buffer at pH 7.4. On the 96-well microplate, 20 µL of Trolox solutions at 0.6, 25, 12.5, 25, 50, and 75 µM as a standard curve, or chlorogenic acid (0.01 mg/mL), or the extracts at a concentration of 25 µg/mL, were then applied. Then, 100 µL of phosphate buffer and 100 µL of extemporaneously prepared fluorescein solution (0.1 µM in phosphate buffer) were added. The microplate was incubated at 37 °C for 10 min with shaking. The reaction was initiated with 50 µL of AAPH. Fluorescence was recorded at an excitation wavelength of 485 nm and an emission wavelength of 535 nm for 70 min using a Tristar LB 941 microplate reader. Final ORAC values were calculated using a regression equation between the Trolox concentration and the area under the curve of the decreasing fluorescein. Data were expressed as micromoles of Trolox equivalents per gram of dry extract.

#### 2.4.2. Immunomodulatory Anti-Inflammatory Bioactivity

##### Macrophage Culture

The macrophage cell line J774.A1 (ATCC, TIB67) was obtained from LGC Standards (LGC Standards, Molsheim, France). Cells were cultured in RPMI 1640 GlutaMAX^®^ medium supplemented with streptomycin (100 µg/mL) and penicillin (100 units/mL), and 10% of inactivated fetal calf serum complete RPMI medium). Cells were incubated at 37 °C, 5% CO_2_, and 95% humidity.

##### Cell Viability by MTS/PMS Assay

To test cytotoxicity, 6.105 cells/well were seeded in a 96-well culture plate in complete RPMI medium, and they were incubated at 37 °C with different concentrations of extracts (25, 50, 75, and 100 μg/mL) for 20 h. After incubation, 20 μL/well of (3-(4,5-dimethylthiazol-2-yl)-5-(3-carboxymethoxyphenyl)-2-(4-sulfophenyl)-2H-tetrazolium), MTS, mixed with an electron coupling reagent, phenazine methosulfate (PMS) in HBSS (Hanks’ balanced salt solution), were added. The plate was incubated for an additional 4 h, and the absorbance at 490 nm was measured in a microplate reader (Thermofisher, Paris, France) as previously described [43].

##### Productions of NO, IL-6, TNF-α, MCP-1, and PGE-2

J774.A1 cells were seeded on a 24-well culture plate with complete RPMI medium. They were pretreated with various concentrations of the OFSP flour extract of 100, 75, 50, and 25 µg/mL for 4 h, stimulated with LPS (100 ng/mL, Lipopolysaccharide *E. coli* 555B5) and murine interferon-gamma (10 ng/mL), and incubated for another 16–18 h at 37 °C. Supernatants were collected for nitrite, PGE-2, TNF-alpha, IL-6, and MCP-1 determination.

##### Determination of Nitrites (NO)

The presence of nitrite, a stable oxidized product of nitric oxide, was determined in the cell culture media as previously described [43]. Briefly, 100 μL of supernatant were combined with 100 μL of Griess reagent in a 96-well plate, and then incubated for 10 min at room temperature. Nitrite concentration was determined by measuring the absorbance at 550 nm using a NaNO_2_ standard curve (1.56 to 100 μM). Results were expressed as a percentage of inhibition values.

##### IL-6 (Interleukin 6) Assay

IL-6 production by J774 cells was determined with the IL-6 ELISA-kit (Mouse IL6 ELISA; Thermo Fisher Scientific, France) after pretreatment with *Ipomoea batatas* flour extract at a determined concentration range (25, 50, 75, and 100 μg/mL) for 18 h. The cells were stimulated with 100 ng/mL LPS (*E. coli*, 555B5) and 10 ng/mL mouse IFN-gamma for 4 h. IL-6 release in cell supernatants was tested according to the ELISA Kit instructions. The results for IL-6 as well as for all other pro-inflammatory cytokines are expressed as a percentage of the inhibition values.

##### TNF-alpha (Tumor Necrosis Factor Alpha) Assay

The tumor necrosis factor alpha (TNF-alpha) assay was performed according to the instructions contained in the kit-ELISA (TNF alpha Mouse Uncoated ELISA kit; Thermo Fisher Scientific, France). After pretreatment with the different concentrations of *Ipomoea batatas* flour extract for 3 h, the cells were stimulated with LPS 100 ng/mL (*E. coli*, 555B5) and mouse IFN-gamma 10 ng/mL for 4 h. TNF-alpha release in cell supernatants was tested by sandwich enzyme-linked immunosorbent ELISA assay.

##### MCP-1 (Monocyte Chemoattractant Protein-1) Assay

Using the ELISA kit (Mouse CCL2 (MCP-1) (Thermo Fisher, Scientific, France), MCP-1 was detected in the cell culture supernatant 18 h after activation with LPS/IFN-gamma. The supernatant was diluted 1:10 in ELISA buffer in order to obtain a concentration of MCP-1 within the calibration range.

##### PGE-2 (Prostaglandin) Assay

The determination of prostaglandins E2 was performed by the competitive enzyme-linked immunosorbent assay (ELISA) on culture supernatants after pretreatment, and the subsequent activation of the cells was performed with LPS/IFN-gamma using the commercial Cayman PGE-2 ELISA KIT Monoclonal.

### 2.5. Statistical Analysis

All statistical analyses were performed using XLSTAT software version 2019.4.1 (Addinsoft, Paris, France). All of the data were reported as means ± standard deviation to the mean (SD) from three replicates of each experiment. Data were analyzed statistically using one-way analysis of variance (ANOVA) in order to determine significant differences (*p* < 0.05). Tukey’s multiple comparison method was used to further examine any significant difference between the results.

## 3. Results

### 3.1. Phytochemical Analysis and Glycemic Index

The physicochemical analysis results of the OFSP flour are presented in Table 1, and contents are expressed as a percentage of the flour dry matter. Flour was obtained with a 35.2% yield and moisture content did not exceed 8.4%, which was lower than the recommended moisture content (13%). Our results indicated a carbohydrate content of 42.7 g/100 g of flour. Starch represented more than 80% of this carbohydrate contribution, with a content of 35.8 g/100 g. Amylose was measured at 14% of total starch. Free sugars were recorded at 6.9 g/100 g while the resistant starch measured after enzymatic digestion for a period of 4 h represented 2.2 g/100 g of flour dry matter, i.e., 6% of the total starch content.

Regarding total fiber content, it is interesting to note that it exceeded 6 g, and that it had interesting rates of cellulose, hemicellulose, and a less important amount of lignin at 1.75 ± 0.01, 4.24 ± 0.02, and 0.21 ± 0.01% flour DM (dry matter), respectively.

The OFSP flour glycemic index was measured in vivo using a glucose solution as reference. OFSP demonstrated a GI of 71, i.e., with a 29% lower GI than the glucose reference (100).

### 3.2. Identification and Quantification of Bioactive Phytochemicals

The phytochemical analysis of the flour extract is presented in Table 2. The mineral content is expressed in g/100 g of dry matter (% DM) and beta-carotene content in mg/100 g (% DM), and the total amount of polyphenols is expressed in mg GAE/g of dry matter (DM).

Determination of mineral matter using the Van Soest method [40] indicated a relatively high amount that could exceed 5.9 g/100 g of flour.

Beta-carotene, measured after extraction, also reached a quantity of 21.8 mg, while the TPC results confirmed the presence of polyphenolic compounds with a value of 38.89 GAE/g EDW (extract dry weight).

### 3.3. Antioxidant Bioactivity

Results obtained with nitric oxide, DPPH, and ORAC scavenging results are presented in Table 3. NO scavenging capacity is expressed as a percentage of inhibition relative to the various concentrations of the flour extract (100, 75, and 50 µg/mL). The ORAC test is expressed in µmol TE/g EDW while DPPH results are represented in µmol TE/g EDW as well as in the percentage of free radical inhibition by an extract concentration of 1 mg/mL.

NO scavenging evaluation demonstrated OFSP extract antioxidant potential expressed in the percentage of inhibition of free radical nitric oxide as a function of the increasing concentration of the extract. Indeed, the concentration-dependent inhibition of NO reached 5%, 15%, and 21.67% at 50, 75, and 100 µg/mL, respectively. DPPH assay corroborated these results with a percentage of inhibition reaching 39.8% at 1 mg/mL of OFSP, which corresponded to 56.9 µmol TE/g of the dry extract. The ORAC test also confirmed the antioxidant capacity of OFSP with a value exceeding 988 µmol TE/g of the dry extract.

### 3.4. Immunomodulatory Anti-Inflammatory Bioactivity

Anti-inflammatory bioactivity tests were performed through the evaluation of OFSP (100, 75, 50, and 25 µg/mL) inhibition of the production of inflammation mediators (NO, TNF-alpha IL-6, MCP-1, and PGE-2) by stimulated macrophages.

The exposure of cells to the OFSP flour extract did not alter the viability of macrophages and this confirmed the non-toxicity of the extracts at the various concentrations that were tested. This result ruled out the potential hypothesis that a decrease in inflammation marker(s) levels could be associated with potential cytotoxicity or with a lower cell number.

**Figure 1:**
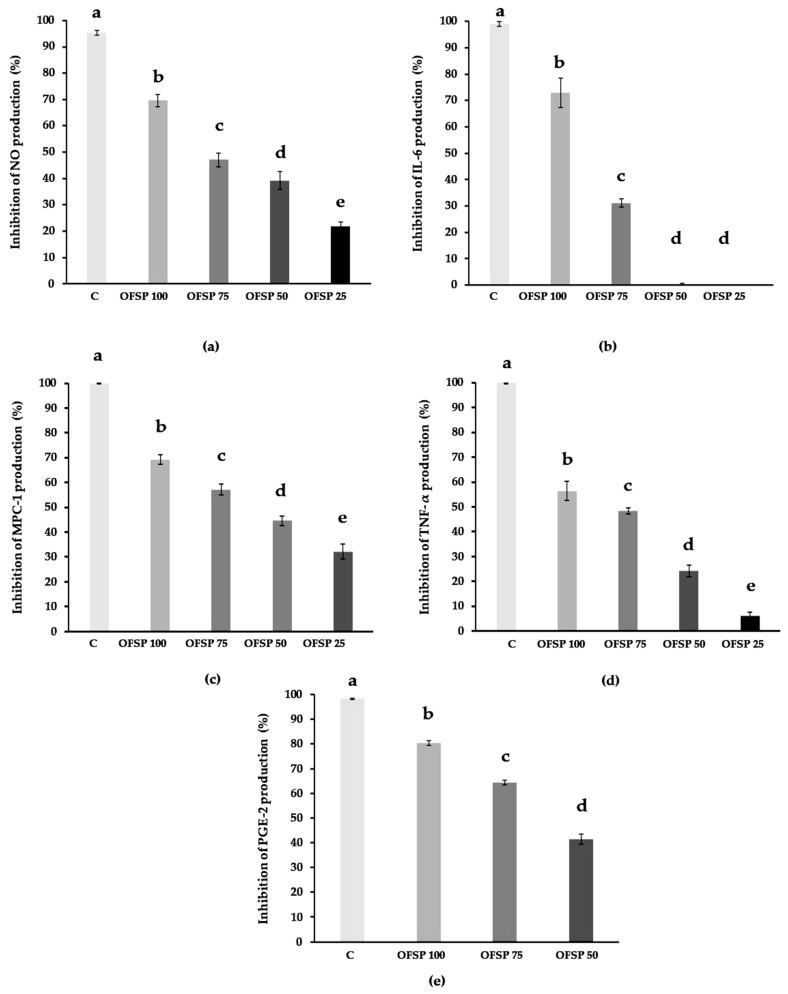
Effect of OFSP flour extract at different concentrations (100, 75, 50, and 25 µg/mL) on NO production (**a**) and on the production of proinflammatory cytokines IL-6 (**b**), MPC-1 (**c**), TNF-alpha (**d**), and PGE-2 (**e**) by J 774 inflammatory macrophages. Histogram bar “C” represents the control value, i.e., macrophage production of NO or cytokine, induced by lipopolysaccharide and interferon-gamma without pre-treatment by OFSP flour extracts. Values measured by Griess/Elisa reagent are expressed as the mean of the percentage inhibition plus or minus the standard deviation (*n* = 3) (a, b, c, d, and e; *p* < 0.05).

The results are shown in Figure 1, and they are expressed as a percentage of inhibition compared to the non-pretreated stimulated control cells.

Nitric oxide (NO) production results are shown in Figure 1a. The graph represents NO production inhibition levels under increasing extract concentrations (25 to 100 µg/mL). Inhibition was statistically significant, ranging from 22 to 69.6%. This observation indicates the OFSP extract concentration-dependent inhibitory bioactivity on pro-inflammatory NO radical production by stimulated macrophage cells. This effect was recorded, and it was significant at all of the concentrations that were tested.

The results obtained with interleukin-6 (IL-6) production, a pro-inflammatory cytokine involved in the acute phase of inflammatory processes, are shown in Figure 1b. The concentration-dependent inhibition of IL-6 production was recorded from concentrations of 50 to 100 µg/mL of OFSP extract. Statistical differences were confirmed between three concentrations (50, 75, and 100 µg/mL). At the two highest concentrations (75 and 100 µg/mL), our results indicate marked inhibitions reaching 31% and 73%, respectively. However, concentrations of 25 and 50 µg/mL induced low levels of inhibition (almost non-existent for the 25 µg/mL dose), with no statistical differences between them.

Thus, it could be demonstrated that concentrations exceeding 75 µg/mL of OFSP extract were associated with a significant level of inhibition of IL-6 production.

Results on monocyte chemotactic protein-1 (MCP-1) production are presented in Figure 1c, which represents the levels of inhibition of this cytokine production according to increasing concentrations of OFSP extract from 25 to 100 µg/mL. Statistical differences were recorded between the percentages of inhibition at the four concentrations. Inhibition reached 57% and 69% at 75 and 100 µg/mL, respectively, while 32% and 45% were recorded at 25 and 50 µg/mL, respectively. This observation indicated a concentration-dependent inhibitory bioactivity of OFSP flour extract on the production (by stimulated macrophages) of MCP-1, which is a key chemokine for the regulation of inflammation development via monocyte/macrophage migration/infiltration processes.

Results obtained on the inhibition of tumor necrosis factor alpha (TNF-alpha) production are shown in Figure 1d. Statistical differences were recorded between the four concentrations tested (25, 50, 75, and 100 µg/mL) with TNF-alpha production inhibition ranging from 6 to 56%. This observation indicated a concentration-dependent inhibitory bioactivity of the OFSP flour extract on the production, by stimulated macrophage cells, of pro-inflammatory cytokine TNF-alpha, which is involved in the acute phase and systemic inflammation processes.

Prostaglandin E2 (PGE-2), i.e., inhibition of production results, as a function of increasing concentrations of the extract (50, 75 and 100 µg/mL), are shown in Figure 1e. Statistical differences were recorded between the three concentrations, with inhibition exceeding 80% at 100 µg/mL, 64% at 75 µg/mL, and 41% at 50 µg/mL. This observation indicated a concentration-dependent inhibition of PGE-2 production, which is a major prostanoid that is involved in the homeostasis of inflammatory processes.

## 4. Discussion

The present study was conducted to explore the phytochemistry and the nutritional potential of flour and extracts obtained by green extraction from orange-fleshed sweet potato (OFSP). The objective of this work was to expand OFSP knowledge in view of its putative use for the management of metabolic diseases associated with oxidative stress and low-grade inflammation.

This root vegetable is one of the most important food crops in the world. It is grown in more than 100 countries in tropical, subtropical, and temperate climates. It is a major staple food in Africa, Asia, the Caribbean, and South America [44]. For several decades, this root was considered as a food security crop. It helps to reduce malnutrition, particularly in sub-Saharan African countries. Sweet potato is usually consumed freshly cooked. However, in an environment where it contributes to food security and limits deficiencies, processing OFSP into dry products can ensure better accessibility and availability through stabilization.

To date, the literature that reports on the physicochemical composition and the biological activities of OFSP indicates great variability, which appears to be associated with the type of accession and extraction modes. However, data on food flour bioactivity are scarce, and the extracts tested are in most cases made from fresh material. We therefore studied the physicochemical composition of OFSP flour obtained by green process in relation to its energetic and nutritional potential. Starch and free sugars, amylose, fibers, and resistant starch were all investigated for their potential positive impact on glycemic and metabolic profiles. Subsequently, we studied the OFSP flour and extract content in bioactive compounds. Indeed, phytochemistry and bioactivity can be modified by the production/extraction process, particularly polyphenols and beta-carotene compounds whose relationship with antioxidant and anti-inflammatory effects are established [45,46].

In our study, a green extraction process (green chemistry) was used to produce OFSP flour and extracts. The green extraction of bioactive natural products is meant to contribute to the protection of the environment, biodiversity, and humanity, and it is based on avoiding petroleum, toxic solvents, and excessive energy whilst using renewable natural resources. It also aims at reducing the number of process unit operations as well as the amount of reagents used, but it also aims to preserve the integrity of natural bioactive compounds. The process and reagents that we used in our investigation are in accordance with the six principles of green extraction. We used natural solvents (alcohols), low-energy consumption mechanical grinding, and air flow drying [47].

As expected, phytochemistry results indicated that carbohydrates were the main flour component. The content of free sugars reached 6.9% of the flour. These results are in agreement with the literature [48]. Total flour starch was recorded at 35.8%. This amount is relatively low when compared to reports from previous studies conducted on flours. Chen’s study of three sweet potato varieties indicated contents ranging from 57.8% to 75.9% [49]. Other investigations performed on flour obtained by a drying process reported contents varying between 64% and 70.2% [50]. Regarding resistant starch, our results are in line with the values from the literature: our data indicate 2.2 g per 100 g of flour, which is equivalent to 6.14 g per 100 g of starch. Previous reports on three batches of OFSP recorded contents of 13.46%, 6.46%, and 1.76% per 100 g of starch [50,51,52]. On the other hand, contents between 11.7% and 4.7% of flour were also measured [50]. As for amylose contents, the literature reports values ranging from 13.3% to 35.5% of total starch [50,51,52]. These contents are higher than the ones that we measured in the present study (5.1%). Comparing these results to previous reports tends to suggest there is a wide range of starch yield for this plant source. Indeed, the literature discusses and mentions several factors that can affect starch yield. Among the most cited are plant source, genetics, varietal differences, environmental pedoclimatic conditions, genetics, and processing steps [53], as well as root storage. Indeed, the latter leads to physiological deterioration that decreases starch content [54,55]. The status of roots used for extraction is also associated with the yield variation; the fresher and better matrix condition, the higher the yield. All of these factors can be controlled. Therefore, careful selection of parameters may lead to starch yield optimization. However, it should be noted that a high yield does not necessarily mean that the starch would have the desired quality for all applications.

Glycemic index (GI) represents a criterion for ranking carbohydrate-containing foods. Numerous studies have shown the importance of this parameter with regards to cardiovascular disease, diabetes, and obesity development and prophylaxis [56,57]. A Japanese study on a large population cohort indicated that diets with a high dietary glycemic load increase the risk of type 2 diabetes [58]. Indeed, high-GI diets were reported to stimulate de novo lipogenesis and lead to increased adipocyte size, whereas low-GI diets inhibit these responses. In addition, diets with low GI reduce postprandial glucose and insulin responses, improve lipid profiles, and increase insulin sensitivity [59]. Strategies that aim to reduce the GI of foods and meals are part of the global approach to prevent diabetes, obesity, and their cardiovascular comorbidities. GI is influenced by starch structure, physical structure, vegetal matrix processing, and the nature and the proportion of other macronutrients present in food. For instance, fat intake decreased the GI of white bread (and thereby glycemic response) in a dose-dependent manner [60]. This may be explained by the fact that amylose-lipid complexes may form, slow down starch digestion, and delay gastric emptying, thereby leading to a lower glycemic response [61].

Other studies on resistant starch and amylose confirm the reduction of postprandial glycemia, insulin response, and therefore GI. The study by Zenel and Stewart [62] reports a significant decrease in the latter following consumption of amylose and resistant starch rich rice compared to rice low in these two components. Similarly, another study highlighted that breads rich in amylose and resistant starch induced lower post-prandial glycemic and insulin response by 39% and 24%, respectively, compared to conventional wheat breads [63]. The levels recorded in our study indicate relatively low amounts of amylose and resistant starch in our flour. Nonetheless, OFSP flour generated a reduced GI (when compared to glucose), i.e., it generated a slow rise of glycemia upon administration. In addition, the glucose load was reduced, with glycemia never rising above 122 mg/dL, and it was thus significantly lower than the glucose reference that reached 168.83 mg/dL, thereby limiting hyper-insulinemic response. Several other macromolecules can have an impact on GI, particularly lipids and/or proteins, the presence of which is negatively associated with the increase in postprandial glycaemia [64].

Dietary fiber is known to play a role in the regulation of the glycemic response and insulinemic response, which would both be associated with low GI. Indeed, our results indicate a content of 6.2 g/100 g. This result completely corroborates with the contents reported by the previous studies [65]. Fujii et al. [66] describe a negative association between fiber intake and glycemic response but also between body mass index, triglycerides, and high sensitivity C-reactive protein. Thus, increased dietary fiber intake was shown to be associated with better glycemic control, lower glycemic index, and better prevention of cardiovascular disease risk factors [56,57].

Numerous works on OFSP describe it as one of the best sources of beta-carotene. It may contain up to 63 times more beta-carotene than non-orange sweet potatoes. Levels of beta-carotene differ among varieties, but reported levels range from 3.76 to 31.45 mg/100 g dry weight. Our study recorded 21.81 mg/100 g. Our results are thus quite comparable to the literature [67]. Beta-carotene is known for its provitamin A activity. Its levels in OFSP make it a food of interest for the improvement of food systems. Carotenoids were shown to exert protective effects against macular degeneration, cardiovascular disease, mutagenesis, and tumor formation [68]. In addition, a cross-sectional study on 108 obese nondiabetic patients revealed an inverse correlation between plasma levels of provitamin A carotenoids and insulin resistance as well as a positive association between plasma beta-carotene and adiponectin concentrations. These results suggest a favorable effect of beta-carotene on insulin sensitivity in obese individuals [69].

The polyphenol content of our sweet potato flour extract was 38.89 mg GAE/g extract. These values are similar to the results that have been previously reported [70] on three sweet potato varieties (38.43, 26.14, and 14.37 mg GAE/g extract).

Regarding the results of our mineral matter determinations, the amount is 5.9% in OFSP flour. This content is relatively high compared to the data in the literature. Indeed, previous results [51] on the starch content of 11 sweet potato varieties varied between 0.10% and 0.47%, while the results of other studies [71] on sweet potato root flours indicate a content between 0.15% and 2.09%. However, these works were conducted on peeled sweet potato roots while our flour is made from dried and ground roots, including the skin. Indeed, previous investigations [72] measured a mineral content approaching 10.1% of DM in sweet potato skins.

Regarding extract antioxidant activity our results demonstrated a dose-dependent nitric oxide (NO) scavenging capacity. NO is a free radical involved in oxidative stress and a pro-inflammatory mediator of acute and chronic inflammation. Furthermore, our DPPH results report a quantifiable and non-negligible antioxidant activity. Previous investigations [70] indicated a relatively similar property (38% inhibition for 1 μg/mL). However, other studies [65] recorded relatively lower activity (2.33 µmol TE/g EDW) compared to our results. These variations may be due to differences in chemical composition potentially attributed to sweet potato genetics, physiology, post-harvest conditions, sample processing, and testing methods. Additionally, ORAC values obtained during our study confirm the antioxidant activity of the extracts. Other works also reported a rather interesting free radical scavenging capacity for this tuber. Notably, a value of 19.6 ± 0.6 μmol TE/g of fresh extract was measured [73,74,75]. These values are very low when compared to our study, but this could be due to the fact that the tested extracts from the literature were made from 5 g of fresh product whereas our extracts were prepared from 50 g of flour. Most studies on OFSP antioxidant properties point in the same direction, indicating an interesting antioxidant activity correlated with its phenolic and beta-carotene content. However, it should be mentioned that variability in raw materials and extraction procedures in addition to genetic and environmental variations induce important variability in the results between investigations [74,75,76,77,78]. The free radical scavenging of OFSP flour extracts may be explained, in part, by their content of phenolic compounds. Indeed, some studies have reported high hydroxycinnamic acid content with quinic and caffeic acid derivatives that are commonly present in various OFSP accessions. Among the most abundant are chlorogenic acid, ferulic acid-o-hexoside, feruoyl quinic acid, and 3,5-di-caffeoyl-quinic acid. In vitro and in vivo experiments have both confirmed caffeic acid and its derivatives as antioxidant, anti-inflammatory, immunostimulatory, antidiabetic, and cardioprotective [79,80]. Other polyphenols were detected in this vegetable root. Specifically, flavonoid kaempferol is abundant in all of the varieties examined [80].

Regarding immunomodulatory anti-inflammatory activity, NO is a nitrogen radical secreted by many cells, particularly in the context of inflammation and immunity [81]. Stimulation of macrophages via an endotoxin (LPS) causes an increased synthesis of NO and inflammatory cytokines. This acute physiological non-specific immune response is designed to counter organism insults. Cytokines and inflammation mediators are also correlated with chronic inflammatory state development, which is itself linked to the development of chronic pathologies development, including certain types of cancers, autoimmune and neurodegenerative diseases, and chronic metabolic diseases, such as type 2 diabetes and cardiovascular diseases [82,83]. In this context, phenolic compounds from plant sources were reported as anti-inflammatory compounds in vitro and in vivo [45,84,85]. In fact, these phytochemicals demonstrated their ability to modulate endogenous anti-inflammatory enzymes, such as catalase, superoxide dismutase (SOD), and glutathione peroxidase [45]. Other works relate their anti-inflammatory power to the modulation of signaling pathways regulating the inflammatory reaction, e.g., NF-kB (Nuclear Factor Kappa-light-chain-enhancer of enabled B cells), MAPk (Mitogen-Activated Protein kinase), PI3K/Akt (Phosphatidylinositide 3-kinase/protein kinase B), PLA2 (phospholipase A2) enzyme, and cyclooxygenases (COX), with a decreased production of prostaglandins [86,87]. Investigations on human keratinocytes have made it possible to study the molecular mechanisms underlying these molecular regulations. The data in the literature indicates a remarkable inhibition of NF-kB activation. In general, all phenolic compounds investigated in this study (i.e., verbascoside, resveratrol, polydatin, quercetin, and rutin) down-regulated the expression of pro-inflammatory cytokine/enzyme genes [88]. Rutin can reduce pancreas inflammation through caspase-1 downregulation and controlled cytokine production [89]. Apigenin (flavonoid) was reported to inhibit the inflammatory response and to decrease cytokines (IL-6, IL-1β, and TNF-alpha) production in J774A.1 murine macrophages. Apigenin inhibited IL-1β production by inhibiting caspase-1 activation. This compound also inhibited TNF-alpha and IL-1β-induced NF-κB activation [90]. Additionally, several reports demonstrated the anti-inflammatory effects of carotenoids [91], including beta-carotene [92]. Similarly, clinical trials demonstrated that the anti-inflammatory properties of carotenoids would be expressed through the modulation of NF-kB and MAPK pathways. Their actions could be synergistic with phenolic compounds [93]. In our study, the production of inflammation mediators by stimulated macrophages was clearly inhibited after treatment with our flour extract. This inhibition was relatively high and concentration dependent. These data are comparable to the literature regarding OFSP. Indeed, many works report an immunomodulatory effect of OFSP extracts measured on activated cells. In their study, Bae et al. (2021) tested an OFSP carotenoids extract on RAW 264.7 cells, and the results indicated a 55% inhibition of NO production for a concentration of 100 μg/mL of extract compared to the control group (treated with LPS only). In the same study, a reduction of IL-6 and PGE-2 production was observed by 99% and 79.1%, respectively. The level of TNF-alpha and IL-1 was nonetheless not significantly reduced when compared with the control group [94].

Our results on OFSP flour extract showed high inhibitions (exceeding 70%) of IL-6 and PGE-2. The lowest inhibition was recorded for the TNF-alpha cytokine (with an inhibition, nevertheless, of 56%). In general, while the results obtained in our work are comparable to previous records [94], it is important to note that the study of Bae et al. was performed using carotenoid extracts and not a flour extract that may contain other compounds, including phenolic compounds. Other work on OFSP supports its dose-dependent anti-inflammatory potential, particularly via the observation of a decrease in inflammatory edema and the inhibition of NO and pro-inflammatory cytokine production (IL-1β and IL-6). The hematological, biochemical, and histological changes noted in this study combined with the inhibitory effects on the production of cytokines recorded during our investigations suggest a preventive and/or therapeutic potential of *Ipomoea batatas* as an anti-inflammatory agent [27].

## 5. Conclusions

The results of our study reveal that, in addition to the basic nutritional effects commonly observed in a root vegetable, the phytochemical composition of orange-fleshed sweet potato flour obtained by green extraction is associated with bioactive properties that are of interest for human health. The nutrient composition indicates a significant contribution to energy, fibers, amylose, minerals, and beta-carotene intake, which may have a combined positive impact on the metabolic and glycemic profile. In addition, evaluation of the biological properties of OFSP flour green extract reveal a significant capacity to reduce oxidative stress as well as inflammation with a dose-dependent inhibition of the production of pro-inflammatory markers, specifically IL-6, TNF-alpha, MCP-1, NO, and PGE-2.

Our results clearly suggest that *Ipomoea batatas* flour green extracts made from sweet potato grown in the southern region of France have antioxidant, anti-inflammatory, and immunomodulatory activities that may contribute to the prophylaxis and/or reduction of the incidence of chronic metabolic diseases.

In view of this, several avenues of investigation can be considered in order to explore its effects in vivo. More specifically, investigations in preclinical animal models of metabolic syndrome with chronic low-grade inflammation or explorations of different transformation processes that could optimize its nutritional and health effects would be of interest. Our research team is engaged in these investigations, and preliminary results are encouraging.

## Figures and Tables

**Table 1:** Physicochemical composition of OFSP flour. Values are expressed as mean plus or minus SD (*n* = 3).

Chemical Composition (%) of OFSP Flour		
**Yield (%)**		35.2
**Moisture** (% flour)		8.4
**Carbohydrates** (% DM flour)		42.7 ± 1.3
	Total starch	35.8 ± 1.8
	Simple sugars	6.9 ± 0.7
**Amylose** (% DM starch)		5.1 ± 0.07
**Resistant starch** (% DM flour)		2.2 ± 0.4
**Total insoluble Fibers** **(% DM flour)**		6.2 ± 0.1
	Hemicellulose	1.75 ± 0.01
	Cellulose	4.24 ± 0.02
	Lignin	0.21 ± 0.01
**Glycemic index** **Maximum glycemia**		71 122.16 ± 2.48 mg/dL

**Table 2:** Composition of orange-fleshed sweet potato flour in mineral matter, beta-carotene, and total amount of polyphenols. Values are expressed as mean plus or minus SD (*n* = 3).

Potentially Bioactive Micronutrient Composition of Orange Sweet Potato Flour			
Sample	TPC in mg GAE/g EDW	Minerals Matters (% DM/g‧100 g^−1^)	beta-Carotene (% DM/mg‧100 g^−1^)
**OFSP**	38.89 ± 0.52	5.93 ± 0.31	21.81 ± 2.20

**Table 3:** Determination of antioxidant potential via NO scavenging, DPPH, and ORAC tests. Values are expressed as mean plus or minus SD (*n* = 3) (a, b, c; *p* < 0,05).

Antioxydant Activity		
Assays		
**NO scavenging**	**Inhibition (%) at 100 µg/mL**	21.67 ± 2.43 ^a^
	**Inhibition (%) at 75 µg/mL**	14.75 ± 2.55 ^b^
	**Inhibition (%) at 50 µg/mL**	4.97 ± 1.80 ^c^
**DPPH**	**µmol TE/g EDW**	56.95 ± 1.73
	**Inhibition (%) at 1 mg/mL**	39.80 ± 1.18
**ORAC**	**µmol TE/g EDW**	988.66 ± 27.49

## Data Availability

Data are contained within the article.
